# Baseline Psychological Factors and Subsequent Exercise Adherence in Individuals with Chronic Low Back Pain

**DOI:** 10.3390/healthcare14213799

**Published:** 2026-11-06

**Authors:** Kyriakos Petropoulakos, Zacharias Dimitriadis, Evdokia Billis, Konstantinos Meliggas, Ioannis Poulis, Savvas Spanos

**Affiliations:** 1 Human Performance and Rehabilitation Research Laboratory, School of Health Sciences, University of Thessaly, 35132 Lamia, Greece; kmeligas@uth.gr (K.M.); ipoulis@uth.gr (I.P.); sspanos@uth.gr (S.S.); 2 Health Assessment and Quality of Life Research Laboratory, University of Thessaly, 35100 Lamia, Greece; zdimitriadis@uth.gr; 3 Faculty of Physiotherapy, School of Health Rehabilitation Sciences, University of Patras, 26504 Patras, Greece; billis@upatras.gr

**Keywords:** chronic low back pain, exercise adherence, psychological distress, kinesiophobia, pain catastrophizing, prospective study

## Abstract

**Highlights:**

**What are the main findings?**

- Higher baseline psychological scores correlated with lower self-reported exercise adherence at Week 8.
- Kinesiophobia showed the most consistent association after adjustment for intervention allocation; catastrophizing and distress findings were less definitive.

**What are the implications of the main findings?**

- Psychological assessment may inform conversations about exercise barriers; the study does not establish that changing a score improves adherence.
- The small sample and self-reported outcome limit interpretation; independent validation is needed before clinical prediction applications.

**Abstract:**

**Background/Objectives**: The independent associations between baseline psychological factors and subsequent exercise adherence in chronic low back pain remain uncertain. This exploratory secondary analysis examined these associations while accounting for intervention allocation. **Methods**: Sixty participants from a randomized trial completed an eight-week exercise program with or without the Move Quiz app. Baseline depression, anxiety and stress were assessed using the Depression Anxiety Stress Scales-21 (DASS-21), kinesiophobia using the Tampa Scale for Kinesiophobia (TSK) and catastrophizing using the Pain Catastrophizing Scale (PCS). Adherence was self-reported using the Exercise Adherence Rating Scale (EARS) at Week 8. Analyses included Spearman correlations, simultaneous regression adjusted for allocation, HC3 robust inference, sensitivity analyses and leave-one-out cross-validation (LOOCV). **Results**: All five baseline scores correlated inversely with EARS (Spearman r_s_ = −0.55 to −0.78; all *p* < 0.001). In the allocation-adjusted model, TSK remained associated with lower EARS (B = −0.228, HC3 95% confidence interval [CI] −0.323 to −0.133; *p* < 0.001). The PCS estimate was negative but less certain (B = −0.082, HC3 95% CI −0.167 to 0.003; *p* = 0.058); DASS-21 subscales showed no clear independent associations. The model including allocation had R^2^ = 0.807, LOOCV R^2^ = 0.757 and root mean squared error = 1.75 EARS points. These are internal performance estimates. **Conclusions**: Kinesiophobia showed the most consistent association with subsequent self-reported adherence. The small sample, self-reported outcome and uncertain catastrophizing and distress findings support cautious interpretation. Statistical associations and internal model performance do not establish causality, clinical utility or accuracy in independently recruited patients.

## 1. Introduction

Low back pain (LBP) is the leading cause of years lived with disability worldwide, affecting approximately 619 million people in 2020, with a projected increase to 843 million by 2050 [1]. Its healthcare, work and socioeconomic consequences are substantial [2]. Chronic low back pain (CLBP), usually defined as symptoms persisting for at least three months, is understood within a biopsychosocial framework [3,4]. Accordingly, rehabilitation addresses physical impairments alongside psychological and social influences [5,6,7]. Exercise is a central component of conservative management, but sustained engagement with the prescribed program remains a practical challenge [8,9]. A 2024 network meta-analysis in adults with nonspecific CLBP found that pain responses varied with exercise modality and dose, with very low to moderate certainty [10]. A separate review of traditional Chinese exercises reported possible pain and disability benefits in middle-aged and older adults with lumbar disc herniation [11]. Its population and exercise content differ from the present nonspecific CLBP sample. These efficacy reviews provide treatment context and do not establish psychological determinants of adherence.

Exercise adherence is influenced by pain experiences, beliefs, motivation, social circumstances and the way treatment is delivered [8,9,12,13]. Reviews identify psychological distress, fear avoidance, kinesiophobia and self-efficacy among potential barriers or enablers [9,12]. Their relationships with adherence are difficult to compare across studies because exercise programs, supervision and adherence definitions differ. Identifying an association with one questionnaire does not establish that it contributes information beyond other related constructs. Fear-avoidance theory proposes that threatening interpretations of pain may encourage avoidance of movement [14]. Self-efficacy concerns confidence in performing a behavior despite barriers [15]; low confidence and low motivation may reduce willingness to initiate or sustain exercise [9,12]. These pathways provide a rationale for studying baseline distress and pain-related cognitions, but motivation and self-efficacy were not measured here, and no mediating mechanism can be tested.

Previous studies have already examined fear avoidance, catastrophizing and psychological distress. In a nine-week stabilization program, Mannion et al. [16] found that exercise self-efficacy was the only psychological or belief-related variable uniquely associated with a multidimensional adherence measure. A case series examining kinesiophobia, catastrophizing, fear avoidance and self-efficacy found no statistically significant cognitive associations, although its sample was very small [17]. Psychometric research using EARS has also reported relationships with psychological and clinical constructs [18]. These findings leave uncertainty about conditional associations with a subsequently measured, standardized adherence outcome.

The present analysis extends this work by examining depression, anxiety, stress, kinesiophobia and catastrophizing together in relation to Week 8 EARS, while accounting for the treatment context. The three distress dimensions describe different emotional symptoms, whereas TSK and PCS assess movement-related fear and catastrophic pain appraisals. Simultaneous entry explores the information attributable to each score conditional on the others; it does not establish five separate mechanisms. Correlation between constructs may reduce precision and destabilize coefficients in a small sample. Exercise self-efficacy was not assessed, despite its previous association with adherence [16]; this analysis therefore cannot establish whether the five scores add information beyond it.

The primary aim was to explore associations between these five baseline scores and adherence at the end of an eight-week program in adults with CLBP. Depression, anxiety and stress were examined separately. We expected higher baseline scores to be associated with lower Week 8 adherence. Multivariable and internal performance analyses were exploratory; they were not intended to produce a clinical prediction rule.

## 2. Materials and Methods

### 2.1. Study Design

This prospective observational secondary analysis used data from a randomized controlled trial in adults with chronic nonspecific low back pain. The parent trial compared the same exercise program performed with Move Quiz, a smartphone application used for attentional distraction (app group) or without it (no-app group). The parent trial evaluated whether adding Move Quiz supported adherence to home exercise. Attentional distraction refers to intentionally directing attention toward an activity other than pain; the application was intended to support this redirection during exercise. This description states its intended function, not a mechanism demonstrated by this analysis. The parent trial was registered at ClinicalTrials.gov (NCT07192809; first posted on 25 September 2025). The present analysis examined associations between baseline psychological scores and subsequent adherence. Ethical approval was obtained from the Internal Ethics Committee of the Department of Physiotherapy, University of Thessaly (approval no. 31123/25/ΤΦΣΚΘ; 14 October 2025). All participants provided written informed consent. The study followed the Declaration of Helsinki [19] and is reported according to STROBE recommendations [20].

### 2.2. Participants and Recruitment

Eligible participants were adults aged ≥18 years with chronic nonspecific low back pain persisting for at least three months. Eligibility was assessed using the inclusion and exclusion criteria of the parent trial. Participants were required to have received no physiotherapy or participated in a structured exercise program during the preceding three months. Exclusion criteria included a history of spinal surgery, neurological conditions affecting movement, regular use of pain-relief medication during the preceding two weeks, pregnancy, and severe psychological disorders that could interfere with participation in the study. For the present study, participants were required to have available baseline data for psychological distress, kinesiophobia, and pain catastrophizing, as well as exercise-adherence data at Week 8. Participants were recruited between November 2025 and April 2026 by convenience and snowball sampling through networks of the University of Thessaly in Lamia, Greece, to facilitate enrolment.

The parent trial enrolled 60 participants, randomly allocated to the app group (*n* = 28) or the no-app group (*n* = 32). All completed the eight-week program and provided complete baseline and Week 8 data for the variables analyzed here; there were no dropouts or exclusions from this secondary analysis. The sample size was fixed by the available parent-trial data, and no separate a priori sample-size calculation was performed for this secondary analysis. With several correlated predictors, this sample may yield imprecise or unstable coefficients and optimistic apparent model fit.

### 2.3. Study Procedure

Following confirmation of eligibility and provision of written informed consent, participants completed the psychological baseline assessment before commencing the intervention of the parent randomized controlled trial. At baseline, psychological distress was assessed using the Depression Anxiety Stress Scales-21 (DASS-21) [21,22,23], kinesiophobia using the Tampa Scale for Kinesiophobia (TSK) [24,25], and pain catastrophizing using the Pain Catastrophizing Scale (PCS) [26]. Depression, anxiety, and stress were considered as separate dimensions of psychological distress.

In the parent trial, participants were randomly allocated to an eight-week exercise program with or without Move Quiz. Both groups used the same strengthening and stabilization program on 2–3 days per week. The program was initially demonstrated, and participants received an individualized exercise plan. Dynamic exercises comprised bilateral straight-leg raises, bilateral prone hip extension, abdominal crunches and prone superman exercises, prescribed for three sets of 8–12 repetitions each. Elastic resistance was individualized when used. Bridge, prone plank and right and left side planks were each prescribed as one isometric hold until fatigue or inability to maintain correct alignment. A side-plank modification with the knees flexed was available. Rest intervals were 30 s between sets and one minute between exercises. Warm-up comprised five minutes of marching in place; cool-down comprised Child Pose, Cobra and Thomas stretches held for 20–30 s each.

Participant-reported exertion was assessed using the Borg CR10 scale (0–10) [27]. Published CLBP exercise protocols describe CR10 monitoring during resistance training [28] and the use of these ratings to guide adjustments to exercise overload [29]. In the present study, the therapist used CR10 ratings to individualize progression according to the published stabilization protocol [29]: ratings of 3–5 prompted an increase in exercise overload, ratings of 6–7 prompted maintenance of the current exercise level, and ratings of 8 or higher prompted a reduction in overload. Exercise difficulty was adjusted through changes in body position and/or elastic-band resistance. For stabilization exercises, progression also included increasing the duration of the isometric hold.

Move Quiz was a self-financed smartphone application used as a concurrent cognitive distraction task in the exercise intervention of the larger parent trial. Throughout the time spent performing the prescribed resistance and stabilization exercises at home, participants engaged with the application’s cognitive distraction task by answering brief questions from several categories: arithmetic calculations, general-knowledge questions concerning capital cities, and questions about colors or familiar features of the environment, such as colors seen in the sky. Participants activated the application by tapping their smartphone and answered the questions verbally. The application received the spoken responses, announced whether each answer was correct or incorrect, and advanced to the next question. The purpose was to engage attention with the question-and-answer task while movement continued, with the intention of diverting attention from pain. The no-app group performed the same exercise program without this concurrent smartphone question task. Task accuracy and the amount of attention directed away from pain were not available in the analysis dataset, so the secondary analysis cannot establish whether the intended attentional mechanism occurred or explained adherence.

Therapist contact with participants took place weekly throughout the eight-week home program. Participation in the home program was monitored through participants’ reports and, in the app group, the Move Quiz logging system. The system recorded the frequency and duration of connections, which the treating therapists could review. These records provided information about application use; connection frequency and duration alone did not verify the number, quality or completeness of exercise sessions. The connection records were not included in the participant-level dataset used for the present secondary analysis. The adherence outcome analyzed here therefore remained the self-reported EARS score collected at Week 8.

### 2.4. Outcome Measures

#### 2.4.1. Depression Anxiety Stress Scales-21 (DASS-21)

Psychological distress was assessed at baseline using the validated Greek DASS-21 [30]. The questionnaire contains three seven-item subscales for depression, anxiety and stress [22,23], with each item scored from 0 to 3. Each seven-item subscale sum was multiplied by two to express scores on the 42-item-equivalent metric (0–42) [21]. During data checking, the investigator confirmed that three entries had inadvertently remained as undoubled raw sums: depression in analysis rows 2 and 15 and stress in row 15. Each of these entries was corrected from 3 to 6 before all analyses reported here. Depression, anxiety and stress were analyzed separately as continuous scores. In a Greek community validation study, Cronbach’s α was 0.85 for depression and 0.84 for both anxiety and stress. Factor analysis supported the three-factor structure, and expected relationships with negative emotions and well-being measures supported construct validity [30]. These are published validation estimates. Individual item responses were unavailable for independent score reconstruction or calculation of internal consistency in the present sample.

#### 2.4.2. Tampa Scale for Kinesiophobia (TSK-GR)

Kinesiophobia was assessed at baseline using the validated Greek version of the Tampa Scale for Kinesiophobia (TSK-GR) [31]. The TSK is a 17-item self-reported questionnaire designed to assess pain-related fear of movement and reinjury [24,25]. Items are rated on a four-point Likert scale, yielding a total score ranging from 17 to 68, with higher scores indicating greater kinesiophobia. The Greek version demonstrated satisfactory internal consistency (Cronbach’s α = 0.74), test–retest reliability (intraclass correlation coefficient [ICC] = 0.78), and construct validity in individuals with CLBP [31].

#### 2.4.3. Pain Catastrophizing Scale (PCS)

Pain catastrophizing was assessed at baseline using the validated Greek version of the Pain Catastrophizing Scale (PCS) [32]. The PCS is a 13-item self-reported questionnaire designed to assess catastrophic thoughts and feelings associated with pain [26]. Each item is rated on a five-point scale ranging from 0 (“not at all”) to 4 (“all the time”), yielding a total score ranging from 0 to 52, with higher scores indicating greater pain catastrophizing. The PCS encompasses three dimensions—rumination, magnification, and helplessness [26]. In the present study, the PCS total score was used as the predictor variable. The Greek version has demonstrated satisfactory reliability and validity in individuals with chronic neck and low back pain [32].

#### 2.4.4. Exercise Adherence Rating Scale (EARS-GR)

Exercise adherence was self-reported once at the end of Week 8 using the standard six-item Greek-language EARS [33,34]. The standard six-item EARS uses responses from 0 to 4; items 1, 4 and 6 are reverse-scored before summation (total 0–24; higher scores indicate greater self-reported adherence) [34]. Its original validation in CLBP reported Cronbach’s α = 0.81 and ICC = 0.97 [34], whereas the Greek validation in older adults reported α = 0.92 and ICC = 0.90 [33]. The printed instructions ask respondents to consider exercises prescribed as part of their treatment, without specifying a recall interval. Accordingly, the outcome is a Week 8 self-report of exercise behavior rather than a defined retrospective assessment of the entire eight-week program.

### 2.5. Statistical Analysis

All descriptive statistics, correlations, regression estimates, sensitivity analyses and internal performance results in this revised report were recalculated from the corrected participant-level analysis file using Python 3.12.14 with NumPy 2.3.5 and SciPy 1.17.0. Continuous variables were summarized using means and standard deviations (SDs), medians and interquartile ranges (IQRs), and observed ranges. Categorical variables were summarized using frequencies and percentages. Distributions were assessed using histograms, normal Q–Q plots and the Shapiro–Wilk test. Baseline psychological scores were also summarized separately by allocation group.

Bivariate associations between the five baseline psychological scores and Week 8 EARS were examined using Spearman’s rank correlation coefficients (r_s_). The 95% confidence intervals (CIs) were obtained from 5000 paired participant-level bootstrap resamples, using the same resample indices for all five correlations (random seed 20261101). Two-sided *p*-values were calculated using Spearman correlation tests.

Four nested linear regression models used Week 8 EARS as the outcome and the same 60 participants. Model 1 included DASS-21 depression, anxiety and stress and TSK; Model 2 added PCS, giving the psychological-only model. Model 3 added intervention allocation (app = 1, no app = 0); Model 4 further added age and sex (male = 1, female = 0). Predictors were entered simultaneously within each model. Model 3 is the principal focus of interpretation because it estimates psychological associations conditional on allocation in a sample with substantial adherence differences between groups. This reporting emphasis was adopted during revision and was not prespecified in the parent trial. All models remain exploratory secondary analyses. Models 1 and 2 provide reduced and psychological-only comparisons; Model 4 assesses sensitivity to additional demographic adjustment.

The contribution of added blocks was described using ΔR^2^ and conventional partial F tests, with HC3 Wald F tests as robust checks. Internal performance was evaluated in 60 leave-one-out cross-validation (LOOCV) folds, predicting each participant’s EARS from the other 59 records. Cross-validated R^2^ was 1 − PRESS/TSS, where PRESS is the sum of held-out squared errors, and TSS is the outcome sum of squares about the full-sample mean; RMSE was √(PRESS/60), in EARS points. The mean-only reference used each training-fold mean. LOOCV evaluates these fixed model specifications internally; it does not establish external accuracy or account for uncertainty in the revision-stage choice of reporting emphasis. These analyses are separate from the parent trial’s treatment-effect analysis.

Principal coefficient inference used heteroscedasticity-consistent HC3 standard errors, with two-sided t tests and 95% confidence intervals based on the residual degrees of freedom. Conventional ordinary least-squares (OLS) results are labeled separately. Diagnostics for the allocation-adjusted model included residual plots, residual Shapiro–Wilk testing, variance inflation factors (VIFs), the studentized Breusch–Pagan (Koenker) test and Cook’s distances. Bootstrap sensitivity analyses for Models 2 and 3 used 10,000 paired participant-level resamples (percentile intervals; random seed 20261101). Model 3 was also refitted after exclusion of observations exceeding Cook’s distance 4/n and in a stability analysis excluding the two participants whose DASS entries had been corrected. All 60 records were retained in the principal analysis. Influence exclusions were data-dependent and exploratory, not a basis for selecting a preferred result.

Regression coefficients were reported as unstandardized B and standardized β, with labeled conventional or HC3 standard errors, confidence intervals and *p*-values. Overall fit was described using F, R^2^ and adjusted R^2^. Tests were two-sided, with *p* < 0.05 as the descriptive significance threshold. Multiple correlations and exploratory models were not multiplicity adjusted; interpretation emphasizes estimate uncertainty and consistency rather than isolated threshold crossings.

Histograms with fitted normal curves, normal Q–Q plots, and Shapiro–Wilk test results supporting the normality assessments are provided in Supplementary File S1.

## 3. Results

### 3.1. Participant Characteristics and Score Distributions

The analysis included 60 participants: 28 in the app group and 32 in the no-app group. Baseline psychological scores, Week 8 EARS, age, sex and allocation were complete for all 60 records. Baseline psychological scores were lower in the app group across all five constructs (Table 1). All participants had tertiary education, as confirmed by the investigator. Diagnosis and education were provided by the investigator rather than recorded individually in the analysis file.

The EARS mean at Week 8 was 9.79 (SD 2.75) in the app group and 4.38 (SD 1.91) in the no-app group; these are descriptive, unadjusted values. Across all participants, the EARS mean was 6.90 (SD 3.58), with a median of 7.0 (interquartile range [IQR] 4.0–10.0) and an observed range of 0–18 (Table 2).

DASS-21 depression, anxiety and stress, and PCS departed from normality (all Shapiro–Wilk *p* < 0.001), as did EARS (*p* = 0.005). TSK did not show a statistically significant departure (*p* = 0.086; Supplementary File S1).

### 3.2. Baseline Associations with Later Exercise Adherence

**Figure 1:**
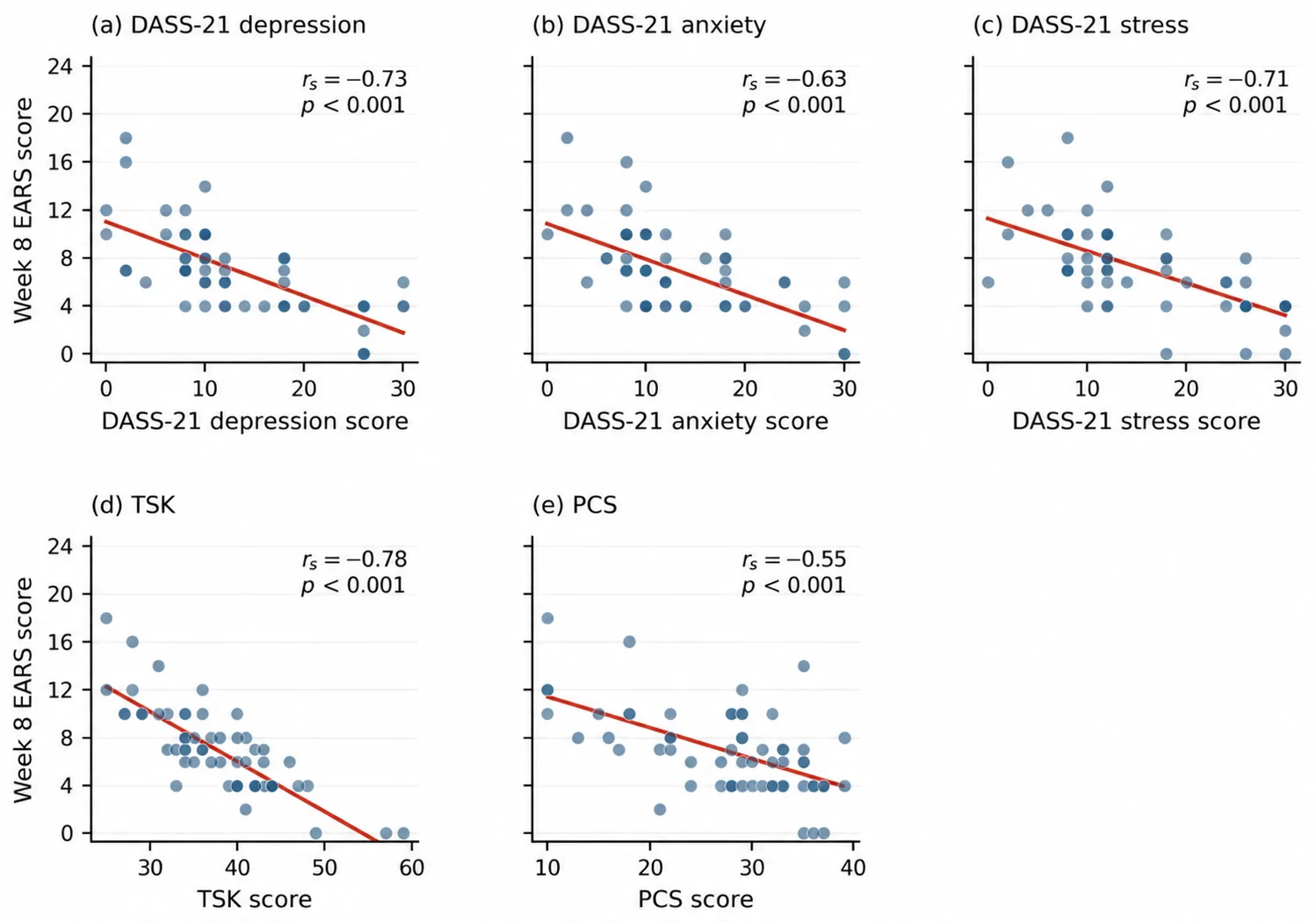
Baseline Depression Anxiety Stress Scales-21 (DASS-21), Tampa Scale for Kinesiophobia (TSK), and Pain Catastrophizing Scale (PCS) scores versus Week 8 Exercise Adherence Rating Scale (EARS) scores (*n* = 60). Red lines show unadjusted ordinary least-squares regression fits; r_s_ denotes the Spearman rank correlation coefficient. Participants with identical score pairs overlap.

All five baseline scores showed inverse bivariate associations with subsequent EARS (Table 3; Figure 1). Spearman coefficients were −0.73 for depression, −0.63 for anxiety, −0.71 for stress, −0.78 for TSK, and −0.55 for PCS (all *p* < 0.001). Higher baseline scores were associated with lower self-reported exercise adherence at Week 8.

### 3.3. Comparison of Nested Regression Models

All four exploratory models used Week 8 EARS and the same 60 participants. Model numbers indicate increasing complexity rather than a prespecified hierarchy of clinical prediction models. Interpretation focuses on the allocation-adjusted Model 3; Models 1 and 2 show the psychological-only comparisons, and Model 4 adds age and sex.

Model 1 included DASS-21 depression, anxiety, and stress scores and TSK. This reduced four-predictor model was statistically significant (F(4, 55) = 32.66, *p* < 0.001), with R^2^ = 0.704 and adjusted R^2^ = 0.682. TSK was associated with lower EARS (B = −0.294, *p* < 0.001), whereas the DASS-21 coefficients did not reach statistical significance (Table 4).

Model 2, containing the five psychological scores without allocation, explained 74.2% of observed EARS variance (R^2^ = 0.742; adjusted R^2^ = 0.718; F(5, 54) = 31.09, *p* < 0.001). TSK and PCS had negative coefficients under conventional and HC3 inference (Table 5). Depression and anxiety did not show clear conditional associations; stress was uncertain (conventional *p* = 0.057; HC3 *p* = 0.067). Adding PCS to Model 1 increased R^2^ by 0.038 (partial F(1, 54) = 8.05, *p* = 0.006). This psychological-only comparison does not establish the PCS association after accounting for allocation.

In LOOCV, Model 1 had R^2^ = 0.649 and RMSE = 2.10 EARS points, compared with R^2^ = 0.681 and RMSE = 2.00 for Model 2. The cross-validated R^2^ increased by 0.032 and RMSE decreased by 0.10 points after adding PCS. A reference model that predicted each held-out participant’s EARS score using the mean score of the remaining 59 participants had an RMSE of 3.61 EARS points. These results describe internal performance and do not establish performance in independent patients.

In the allocation-adjusted Model 3, TSK remained associated with lower EARS (B = −0.228 per TSK point; HC3 95% CI −0.323 to −0.133; *p* < 0.001). The PCS estimate remained negative, but its HC3 interval included zero (B = −0.082; 95% CI −0.167 to 0.003; *p* = 0.058), whereas its conventional *p*-value was 0.013. The DASS-21 subscales showed no clear independent associations. The app coefficient was +2.50 EARS points (HC3 95% CI 1.55 to 3.45; *p* < 0.001). R^2^ was 0.807 and adjusted R^2^ 0.785; adding allocation increased R^2^ by 0.065 (partial F(1, 53) = 17.88, *p* < 0.001). LOOCV R^2^ was 0.757, and RMSE was 1.75 points. These fit measures concern psychological scores plus allocation and cannot be attributed to psychological scores alone (Table 6).

Model 4 added age and sex to the allocation-adjusted model (Table 7). R^2^ was 0.808 and adjusted R^2^ was 0.778. Their joint addition did not improve fit (ΔR^2^ = 0.001; partial F(2, 51) = 0.12, *p* = 0.886). TSK remained associated with EARS under HC3 inference, whereas PCS remained uncertain (HC3 *p* = 0.070). LOOCV R^2^ was 0.741, and RMSE was 1.81 points. This sensitivity model did not improve internal performance over Model 3. Allocation coefficients in either model do not replace the parent trial’s treatment-effect analysis.

The sequential model specifications, incremental fit statistics, and internal predictive performance results are summarized in Table 8.

### 3.4. Regression Diagnostics and Sensitivity Analyses

**Figure 2:**
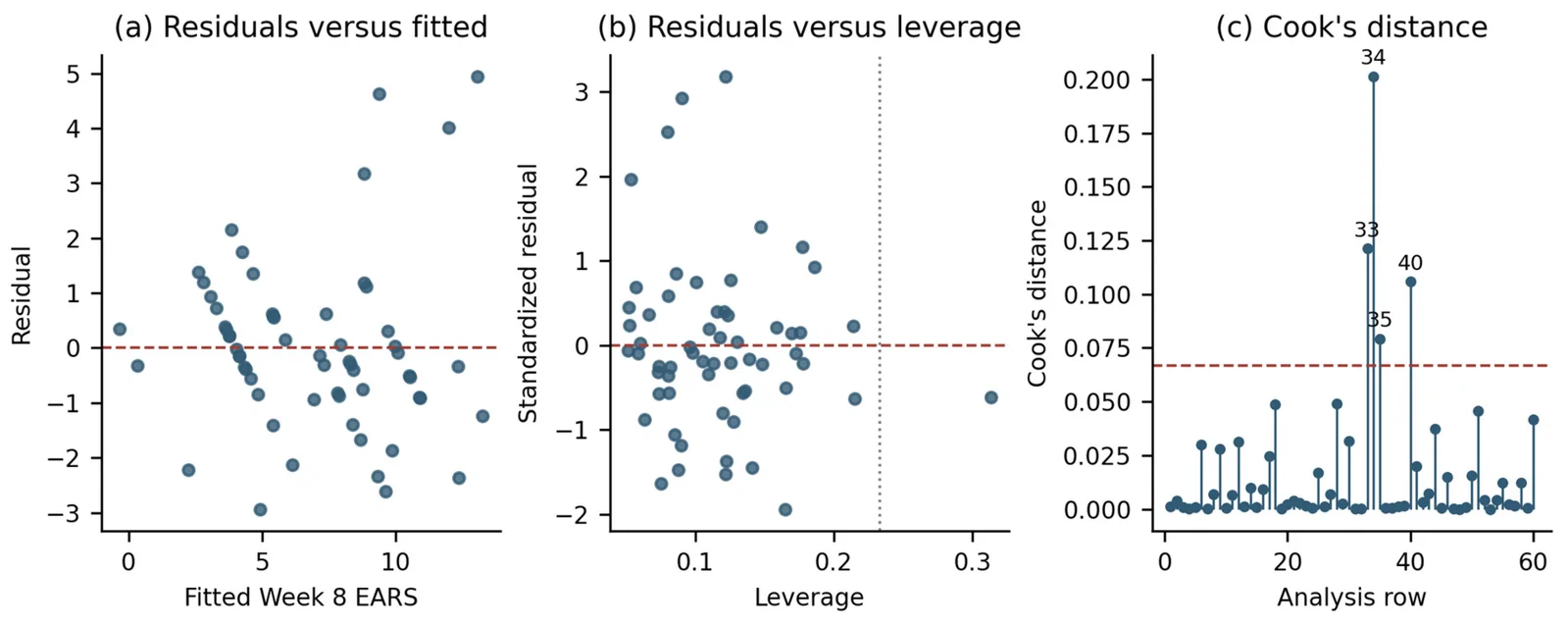
Diagnostics for the allocation-adjusted Model 3 (*n* = 60). Panels show (**a**) residuals versus fitted EARS, (**b**) internally standardized residuals versus leverage, and (**c**) Cook’s distances by analysis row. Dashed horizontal lines mark zero residuals in (**a**,**b**) and 4/n in (**c**). The dotted vertical line in (**b**) marks leverage 2(k + 1)/n, where k = 6. Row numbers indicate positions in the analysis table, not participant identifiers.

Diagnostics for the allocation-adjusted Model 3 showed non-normal residuals (Shapiro–Wilk *p* < 0.001). The studentized Breusch–Pagan (Koenker) test did not detect heteroscedasticity (LM = 7.82, *p* = 0.251; auxiliary F(6, 53) = 1.32, *p* = 0.263). VIFs ranged from 1.38 to 3.22. 4 observations exceeded Cook’s-distance 4/n (analysis rows 33, 34, 35 and 40; maximum 0.201); none exceeded 1. All observations were retained in the principal analysis (Figure 2).

Excluding the four observations flagged by Model 3 gave *n* = 56 and R^2^ = 0.866. TSK remained negatively associated with EARS (B = −0.193; HC3 *p* < 0.001); PCS also excluded zero under HC3 inference (B = −0.089; *p* < 0.001). This data-dependent exclusion is exploratory. In a separate stability analysis, excluding the two participants whose DASS entries had been corrected gave *n* = 58 and R^2^ = 0.799. TSK remained associated (B = −0.229; HC3 95% CI −0.328 to −0.131; *p* < 0.001), while PCS remained uncertain (B = −0.087; HC3 95% CI −0.183 to 0.008; *p* = 0.073). All principal results use the full sample of 60 participants and the corrected scores.

One in-sample fitted EARS value from Model 3 was below zero (−0.341); none exceeded 24. The psychological-only Model 2 produced 2 fitted values below zero. Linear regression was used to explore conditional associations; these out-of-range values limit its suitability as an individual clinical prediction rule. No clipping or bounded-outcome model was applied.

TSK retained a negative association with HC3 inference in all four models. PCS had HC3 *p* = 0.024 in Model 2, but *p* = 0.058 and 0.070 after allocation adjustment in Models 3 and 4. In Model 3, the paired-bootstrap PCS interval narrowly excluded zero, whereas the HC3 interval included it (Table 9). Thus, PCS inference depended on adjustment, inferential method and influential observations. None of the three DASS-21 subscales had a Model 3 HC3 or bootstrap interval that excluded zero. In the psychological-only Model 2, stress had conventional *p* = 0.057 and HC3 *p* = 0.067, while its bootstrap 95% CI was −0.163 to −0.008. This method-dependent result did not persist after allocation adjustment and is considered exploratory.

The HC3 robust Wald tests for the added predictor blocks are presented in Table 10. These tests supported the addition of intervention allocation to the psychological-only model, whereas the joint addition of age and sex was not supported.

## 4. Discussion

### 4.1. Principal Findings and Pain-Related Cognitions

In this exploratory secondary analysis of 60 adults with CLBP, all five baseline psychological scores correlated inversely with self-reported Week 8 adherence. Kinesiophobia showed the most consistent conditional association after accounting for the other scores and intervention allocation. The PCS estimate remained negative, but its uncertainty depended on the inferential method and influential observations. Depression, anxiety and stress showed no clear independent associations in the allocation-adjusted model. Temporal ordering supports prospective associations, but it does not establish that reducing fear or catastrophic thoughts would improve adherence.

Previous findings are mixed. Mannion et al. [16] identified exercise self-efficacy as the only psychological variable uniquely associated with a multidimensional adherence measure; a small case series found no significant TSK or PCS associations [17]. Dhondt et al. [35] reported that higher kinesiophobia predicted greater completion of outpatient multimodal rehabilitation, in contrast to our negative EARS association. Program completion, sessions performed and EARS scores capture different behaviors, and supervision may change how movement-related concern affects participation. Variation in participant selection and baseline clinical profiles may also contribute to differences between studies. Program duration also differed: Mannion et al.’s program lasted nine weeks, compared with eight weeks here. These differences limit direct comparison.

Fear-avoidance theory provides a plausible interpretation: anticipated movement-related threat may be followed by fear and avoidance [14]. Later accounts also emphasize competing goals and treatment context [36]. Our baseline questionnaires did not measure avoidance during exercise or test a mediating pathway. The TSK association therefore supports further investigation of movement-related fear without demonstrating a mechanism or a treatment target whose modification will improve adherence.

PCS illustrates the distinction between an association and useful prediction. Adding PCS to the reduced four-score model (Model 1) increased LOOCV R^2^ by 0.032 and reduced RMSE by 0.10 EARS points. After allocation adjustment, its HC3 interval included zero, while its bootstrap interval narrowly excluded it. Such method dependence, together with changes after influence exclusions, does not support a definitive independent effect. Adherence reviews describe interacting patient and treatment factors [8,9,12], which the present five scores cannot fully represent.

Self-efficacy is a particularly relevant omission. Confidence in performing a behavior can influence its initiation and persistence [15], and prior CLBP work associated exercise self-efficacy with adherence [16]. Because it was not assessed here, we cannot determine whether TSK or PCS adds information beyond self-efficacy or partly reflects a related unmeasured characteristic.

### 4.2. Psychological Distress and Shared Information

The strong bivariate distress correlations diminished when the scores were entered together. Each regression coefficient represents a conditional association after accounting for the other included variables; shared information between distress, fear and catastrophizing may reduce coefficient precision. This does not demonstrate that distress is irrelevant, nor does it identify which construct explains the shared association. VIFs below conventional concern thresholds do not eliminate coefficient instability in a sample of 60. The allocation-adjusted HC3 and bootstrap results did not provide clear independent evidence for any DASS-21 subscale.

Context also matters. Other secondary analyses have related stress to cognitive behavioral treatment completion [37] and emotional well-being to engagement in movement classes [38]; social support has been associated with exercise sessions over time [39]. These outcomes and time frames differ from Week 8 EARS. They identify plausible additional influences rather than confirm a mechanism in the present data.

### 4.3. Intervention and Digital Context

The app group had a higher unadjusted mean EARS score (9.79 versus 4.38) and lower scores on all five baseline psychological measures. These descriptive differences make allocation adjustment important when interpreting pooled psychological associations. After adjustment, the TSK association persisted, and PCS evidence became less definitive. The allocation coefficient was +2.50 EARS points (HC3 95% CI 1.55 to 3.45). The attentional distraction component may have influenced adherence independently of baseline characteristics, but this secondary model cannot distinguish effects on exercise behavior, experience or self-report, establish mediation, or replace the parent trial’s treatment-effect analysis. Baseline group differences also warrant consideration alongside the original allocation procedures and assessment timing.

Digital interventions vary in content. Lechauve et al. [40] found no six-month EARS difference when a self-management app was added to care, despite some other favorable outcomes; its intervention and assessment period differ from ours. Work on app engagement and patient expectations emphasizes beliefs, clinician introduction and feedback [41,42]. Move Quiz recorded connection frequency and duration for monitoring, but these records and user-experience measures were not included in the present analyses. Their contribution to the group contrast therefore remains untested.

### 4.4. Internal Model Performance

The psychological-only model had apparent R^2^ = 0.742 and LOOCV R^2^ = 0.681 (RMSE = 2.00 EARS points). Including allocation increased these values to 0.807 and 0.757, respectively (RMSE = 1.75). The latter performance includes treatment information and should not be attributed to psychological assessment alone. LOOCV predicts each record using the other 59; it does not establish accuracy or clinical usefulness in an independently recruited cohort, and no threshold for clinically useful prediction error was evaluated.

With correlated predictors, six parameters beyond the intercept in Model 3 and only 60 records, apparent fit may be optimistic and coefficients unstable. Model 4 added complexity without improving internal performance. LOOCV does not remove uncertainty from the exploratory modeling and revision-stage reporting choices. Non-normal residuals and the fitted EARS value below zero further limit individual prediction. Future prediction work should evaluate models respecting the bounded outcome, calibration, external performance and clinical usefulness; these properties cannot be inferred from R^2^ alone.

### 4.5. Clinical Implications

A discussion of movement-related fears and practical exercise barriers may be appropriate when assessing a patient with CLBP. These findings do not establish screening cutoffs, justify labeling someone nonadherent, or show that lowering TSK improves adherence. Qualitative studies emphasize individual circumstances, exercise identity, knowledge of correct performance and self-initiated strategies [43,44]. These issues remain relevant alongside psychological questionnaire scores.

Communication-skills training produced a small improvement in adherence ratings in one trial [45], and goal setting improved rehabilitation adherence in another population [46]. These interventions and measures differ from the present study. They support further study of practical adherence assistance without establishing that the observed TSK or PCS associations identify who will benefit.

### 4.6. Strengths and Limitations

Strengths include prospective ordering of psychological scores and EARS, complete analysis variables, explicit allocation adjustment, robust inference and internal sensitivity checks. The sample was nevertheless small relative to the six predictors in Model 3 and eight in Model 4, and was drawn from one parent trial. Several predictors are correlated, so coefficient precision and stability are limited. Convenience and snowball recruitment through networks of the University of Thessaly in Lamia and the uniformly tertiary-educated sample limit representativeness. Findings may not generalize to people with other educational backgrounds or to different clinical and treatment settings.

The adherence outcome analyzed here was self-reported EARS at Week 8. This permits recall and common-method bias and does not establish how exercise participation changed across eight weeks. Baseline pain intensity, functional disability, exercise self-efficacy, previous exercise behavior, social support and symptom duration were not included in the models; residual confounding remains possible. Although Move Quiz connections were logged for program monitoring, these records were not included in the analyses and could not independently verify exercise completion. Objective verification of completed exercise and questionnaire item responses were unavailable for this analysis. Model 3 residuals were non-normal, four observations exceeded the Cook’s distance screening threshold, and one fitted value was below the EARS minimum. Multiple exploratory analyses and internally assessed performance further limit definitive interpretation.

### 4.7. Future Research

Larger, independently recruited cohorts should test whether the TSK association persists across symptom severity, education and treatment settings. Repeated adherence assessments alongside exercise diaries or device-based records could distinguish uptake from sustained participation. Pain, disability, self-efficacy, prior exercise behavior and social support should be measured, and quantitative app-engagement records should be incorporated into the analyses to assess competing explanations and the incremental information from psychological scores.

Future prediction models should be prespecified, address the bounded EARS outcome and undergo independent calibration and performance assessment. Sample-size planning should consider anticipated explanatory strength, the number of parameters and required precision [47]. A separate randomized study would be needed to determine whether an intervention reducing movement-related fear improves adherence. Prediction and causal intervention questions remain unresolved here.

## 5. Conclusions

In 60 adults with CLBP, higher baseline depression, anxiety, stress, kinesiophobia and catastrophizing correlated with lower self-reported exercise adherence at Week 8. Kinesiophobia showed the most consistent association after adjustment for the other scores and intervention allocation. Evidence for catastrophizing was less definitive, and distress subscales showed no clear independent associations in the allocation-adjusted model. The small sample, self-reported outcome and unmeasured influences require cautious interpretation. These exploratory associations do not establish causality or a clinically applicable prediction model; confirmation in larger, independently recruited samples is needed.

## Figures and Tables

**Table 1:** Participant characteristics and baseline psychological scores by intervention allocation (*n* = 60).

Characteristic	Overall (*n* = 60)	App (*n* = 28)	No App (*n* = 32)
Age, years, mean (SD)	42.02 (13.46)	41.43 (11.55)	42.53 (15.11)
Male, *n* (%)	31 (51.7%)	15 (53.6%)	16 (50.0%)
Female, *n* (%)	29 (48.3%)	13 (46.4%)	16 (50.0%)
Chronic nonspecific low back pain, *n* (%)	60 (100%)	28 (100%)	32 (100%)
Tertiary education, *n* (%)	60 (100%)	28 (100%)	32 (100%)
DASS-21 depression, mean (SD)	13.40 (8.07)	8.64 (4.72)	17.56 (8.14)
DASS-21 anxiety, mean (SD)	13.40 (7.58)	9.14 (4.50)	17.12 (7.82)
DASS-21 stress, mean (SD)	16.37 (8.89)	10.64 (5.25)	21.38 (8.43)
TSK, mean (SD)	37.88 (6.91)	33.50 (5.11)	41.72 (5.97)
PCS, mean (SD)	27.53 (7.93)	23.96 (8.79)	30.66 (5.56)

Age, sex, allocation and psychological scores were extracted from the analysis dataset. Psychological entries are reported as baseline scores in the study records. Diagnosis and education were reported by the investigator. Group summaries are descriptive. SD = standard deviation; DASS-21 = Depression Anxiety Stress Scales-21; TSK = Tampa Scale for Kinesiophobia; PCS = Pain Catastrophizing Scale.

**Table 2:** Descriptive statistics for the complete analytical sample (*n* = 60).

Scale	Mean (SD)	Median [Q1, Q3]	Range
DASS-21 depression	13.40 (8.07)	10.0 [8.0, 18.0]	0–30
DASS-21 anxiety	13.40 (7.58)	10.0 [8.0, 18.0]	0–30
DASS-21 stress	16.37 (8.89)	12.0 [10.0, 26.0]	0–30
TSK	37.88 (6.91)	38.0 [34.0, 42.0]	25–59
PCS	27.53 (7.93)	29.0 [22.0, 33.0]	10–39
EARS	6.90 (3.58)	7.0 [4.0, 10.0]	0–18

DASS-21 = Depression Anxiety Stress Scales-21; TSK = Tampa Scale for Kinesiophobia; PCS = Pain Catastrophizing Scale; EARS = Exercise Adherence Rating Scale; SD = standard deviation; Q1 and Q3 = first and third quartiles.

**Table 3:** Bivariate associations between baseline scores and Week 8 EARS (*n* = 60).

Baseline Scale	Spearman r_s_	95% CI	*p*
DASS-21 depression	−0.73	−0.83 to −0.58	<0.001
DASS-21 anxiety	−0.63	−0.76 to −0.43	<0.001
DASS-21 stress	−0.71	−0.82 to −0.55	<0.001
TSK	−0.78	−0.87 to −0.65	<0.001
PCS	−0.55	−0.73 to −0.32	<0.001

r_s_ = Spearman rank correlation coefficient; CI = confidence interval. Percentile CIs were recalculated from 5000 paired participant-level bootstrap resamples (seed 20261101). All *p*-values are two-sided and unadjusted for the five correlations.

**Table 4:** Model 1: reduced four-score regression model for Week 8 EARS (*n* = 60).

Term	B	OLS SE	β	OLS 95% CI	OLS *p*	HC3 *p*
Intercept	20.385	1.720	—	16.938 to 23.831	<0.001	<0.001
DASS-21 depression	−0.045	0.058	−0.102	−0.162 to 0.071	0.437	0.405
DASS-21 anxiety	−0.024	0.052	−0.051	−0.129 to 0.081	0.648	0.590
DASS-21 stress	−0.086	0.043	−0.213	−0.173 to 0.001	0.052	0.070
TSK	−0.294	0.058	−0.569	−0.411 to −0.177	<0.001	<0.001

B = unstandardized coefficient; SE = conventional OLS standard error; β = standardized coefficient; CI = confidence interval. OLS SEs, CIs and *p*-values use conventional inference. HC3 *p*-values use robust standard errors and 55 residual degrees of freedom. R^2^ = 0.704; adjusted R^2^ = 0.682; F(4, 55) = 32.66, *p* < 0.001.

**Table 5:** Model 2 psychological-only regression for Week 8 EARS (*n* = 60).

Term	B	OLS SE	β	OLS 95% CI	OLS *p*	HC3 *p*	VIF
Intercept	21.921	1.707	—	18.498 to 25.344	<0.001	<0.001	—
DASS-21 depression	−0.028	0.055	−0.063	−0.138 to 0.082	0.616	0.616	3.22
DASS-21 anxiety	−0.005	0.050	−0.010	−0.105 to 0.095	0.922	0.908	2.34
DASS-21 stress	−0.079	0.041	−0.197	−0.161 to 0.003	0.057	0.067	2.15
TSK	−0.276	0.055	−0.533	−0.387 to −0.165	<0.001	<0.001	2.40
PCS	−0.103	0.036	−0.228	−0.175 to −0.030	0.006	0.024	1.35

B = unstandardized coefficient; SE = conventional OLS standard error; β = standardized coefficient; CI = confidence interval; VIF = variance inflation factor. OLS SEs, CIs and *p*-values use conventional inference; HC3 *p*-values use robust standard errors and 54 residual degrees of freedom. R^2^ = 0.742; adjusted R^2^ = 0.718; F(5, 54) = 31.09, *p* < 0.001. Model 2 excludes allocation and is a psychological-only comparison.

**Table 6:** Model 3 allocation-adjusted regression for Week 8 EARS with HC3 inference (*n* = 60).

Term	B	HC3 SE	β	HC3 95% CI	OLS *p*	HC3 *p*
Intercept	17.351	2.118	—	13.103 to 21.599	<0.001	<0.001
DASS-21 depression	−0.031	0.037	−0.070	−0.106 to 0.044	0.521	0.410
DASS-21 anxiety	0.014	0.037	0.031	−0.060 to 0.089	0.742	0.696
DASS-21 stress	−0.031	0.031	−0.077	−0.094 to 0.032	0.414	0.331
TSK	−0.228	0.047	−0.440	−0.323 to −0.133	<0.001	<0.001
PCS	−0.082	0.042	−0.182	−0.167 to 0.003	0.013	0.058
App group	2.497	0.473	0.351	1.550 to 3.445	<0.001	<0.001

B = unstandardized coefficient; β = standardized coefficient; CI = confidence interval. SEs, CIs and HC3 *p*-values use HC3 robust inference with 53 residual degrees of freedom. OLS *p*-values are shown separately for comparison and should not be paired with HC3 CIs as one inferential procedure. App = 1, no app = 0. R^2^ = 0.807; adjusted R^2^ = 0.785; conventional F(6, 53) = 36.99, *p* < 0.001. Model 3 is the principal focus of interpretation in this revised exploratory report.

**Table 7:** Model 4 additionally adjusted for age and sex with HC3 inference (*n* = 60).

Term	B	HC3 SE	β	HC3 95% CI	OLS *p*	HC3 *p*
Intercept	17.367	2.392	—	12.565 to 22.169	<0.001	<0.001
DASS-21 depression	−0.024	0.041	−0.054	−0.106 to 0.059	0.643	0.565
DASS-21 anxiety	0.013	0.038	0.028	−0.062 to 0.089	0.768	0.721
DASS-21 stress	−0.034	0.034	−0.085	−0.103 to 0.035	0.387	0.324
TSK	−0.232	0.050	−0.448	−0.332 to −0.132	<0.001	<0.001
PCS	−0.082	0.044	−0.181	−0.170 to 0.007	0.015	0.070
App group	2.497	0.484	0.351	1.525 to 3.468	<0.001	<0.001
Age (years)	0.005	0.016	0.018	−0.028 to 0.037	0.785	0.770
Male sex	−0.181	0.490	−0.025	−1.164 to 0.802	0.689	0.713

B = unstandardized coefficient; β = standardized coefficient; CI = confidence interval. SEs, CIs and HC3 *p*-values use HC3 robust inference with 51 residual degrees of freedom; conventional OLS *p*-values are labeled separately. R^2^ = 0.808; adjusted R^2^ = 0.778; conventional F(8, 51) = 26.85, *p* < 0.001. Model 4 is a demographic-adjustment sensitivity analysis.

**Table 8:** Sequential specification, incremental fit, and internal predictive performance of nested regression models for Week 8 EARS (*n* = 60).

**A. Model Specification and Incremental Fit**							
**Model**	**Predictors Added**	**k**	**R^2^**	**Adjusted R^2^**	**ΔR^2^**	**F Change (df)**	***p* Change**
1 Reduced	DASS-21 depression, anxiety, stress; TSK	4	0.704	0.682	—	—	—
2 Psychological-only	PCS	5	0.742	0.718	0.038	8.05 (1, 54)	0.006
3 Allocation-adjusted	Intervention group	6	0.807	0.785	0.065	17.88 (1, 53)	<0.001
4 Age and sex	Age and sex	8	0.808	0.778	0.001	0.12 (2, 51)	0.886
**B. Internal Predictive Performance**							
**Model**	**LOOCV R^2^**				**LOOCV RMSE**		
1 Reduced	0.649				2.10		
2 Psychological-only	0.681				2.00		
3 Allocation-adjusted	0.757				1.75		
4 Age and sex	0.741				1.81		

All models used the same 60 records and Week 8 EARS. Each successive model retained all preceding predictors, entered simultaneously. Model 2 is the psychological-only comparison; Model 3, including allocation, is the principal interpretative focus adopted during revision; Model 4 adds age and sex. k excludes the intercept. ΔR^2^ and F change compare successive models using unrounded values. LOOCV is internal evaluation; RMSE is in EARS points. The mean-only LOOCV reference RMSE was 3.61.

**Table 9:** Paired-bootstrap coefficient intervals for allocation-adjusted Model 3 (*n* = 60).

Term	Bootstrap 95% CI
Intercept	13.651 to 21.434
DASS-21 depression	−0.100 to 0.048
DASS-21 anxiety	−0.056 to 0.084
DASS-21 stress	−0.098 to 0.024
TSK	−0.318 to −0.140
PCS	−0.162 to −0.006
App group	1.527 to 3.417

Percentile intervals were obtained from 10,000 paired participant-level resamples (seed 20261101). TSK and allocation intervals excluded zero. The PCS bootstrap interval narrowly excluded zero, whereas its HC3 95% CI included zero (*p* = 0.058); this method dependence supports cautious interpretation. Bootstrap intervals for all three DASS-21 subscales included zero. Bootstrap inference is an internal sensitivity analysis, not external validation.

**Table 10:** HC3 sensitivity tests for the added predictor blocks.

Comparison	Added Block	HC3 Wald F (df)	*p*
Model 1 to 2	PCS	5.43 (1, 54)	0.024
Model 2 to 3	App group	27.93 (1, 53)	<0.001
Model 3 to 4	Age (years), Male sex	0.10 (2, 51)	0.908

Conventional partial F tests compare residual sums of squares and assume independent, homoscedastic errors; HC3 Wald tests provide a robust check for each added block. Both approaches support adding allocation to the psychological-only model. The PCS test compares Models 1 and 2 before allocation adjustment; it should not be interpreted as evidence of a robust PCS association in Model 3. Neither approach supports adding age and sex together.

## Data Availability

The data presented in this study are available on request from the corresponding author due to privacy and confidentiality considerations related to participants’ personal and health data.

## Supplementary Materials

The following supporting information can be downloaded at: https://www.mdpi.com/article/10.3390/healthcare14213799/s1, Supplementary File S1: Score Distributions and Normality Assessment.

## Abbreviations

The following abbreviations are used in this manuscript:

LBP	Low back pain
CLBP	Chronic low back pain
DASS-21	Depression Anxiety Stress Scales-21
TSK	Tampa Scale for Kinesiophobia
PCS	Pain Catastrophizing Scale
EARS	Exercise Adherence Rating Scale
SD	Standard deviation
SE	Standard error
IQR	Interquartile range
CI	Confidence interval
ICC	Intraclass correlation coefficient
VIF	Variance inflation factor
HC3	Heteroscedasticity-consistent covariance estimator, type 3
LM	Lagrange multiplier
LOOCV	Leave-one-out cross-validation
RMSE	Root mean squared error
STROBE	Strengthening the Reporting of Observational Studies in Epidemiology
