# Supplementary material for: Baseline Psychological Factors and Subsequent Exercise Adherence in Individuals with Chronic Low Back Pain
Source: Healthcare (Basel). 2026 Nov 6;14(21):3799. doi: 10.3390/healthcare14213799 (PMC13762561; doi:10.3390/healthcare14213799)
Supplement: Supplementary file 1 [file healthcare-14-03799-s001.zip › healthcare-4638730-supplementary.pdf]

Supplementary File S1. Score distributions and normality assessment

Baseline psychological factors and subsequent exercise adherence in individuals with chronic low back pain

This supplement presents score distributions for all 60 participants: baseline DASS-21 depression, anxiety and stress, Tampa Scale for Kinesiophobia (TSK), Pain Catastrophizing Scale (PCS), and Exercise Adherence Rating Scale (EARS) at Week 8. Histograms, normal Q–Q plots and Shapiro–Wilk results were regenerated from the corrected analysis data.

Following the investigator's confirmation that three DASS-21 entries had not been doubled, depression totals in analysis rows 2 and 15 and the stress total in analysis row 15 were corrected from 3 to 6. These were investigator-confirmed scoring-entry corrections, not reconstructions from item-level questionnaire forms. All other recorded values were retained.

Table S1 Shapiro–Wilk tests for the six score distributions

Scale	n	Shapiro–Wilk W	p value
DASS-21 depression	60	0.918567	0.000671822
DASS-21 anxiety	60	0.903303	0.000173921
DASS-21 stress	60	0.903197	0.000172352
TSK	60	0.965344	0.086119173
PCS	60	0.918891	0.000692198
Week 8 EARS	60	0.939804	0.005307051

Values are reported numerically rather than as significance thresholds. DASS-21 depression, anxiety and stress, PCS and EARS departed from a normal distribution at $p < 0.05$; TSK did not show a statistically significant departure ($p = 0.086119173$). These tests concern marginal score distributions and do not establish the normality of regression residuals.

In Figures S1–S3, histogram areas are normalized to density. Solid curves show normal densities fitted using the sample mean and sample standard deviation. Q–Q plots compare ordered recorded scores with theoretical normal quantiles; dashed lines are fitted reference lines. Questionnaire scores are discrete, so repeated scores produce horizontal steps.

DASS 21 depression and anxiety

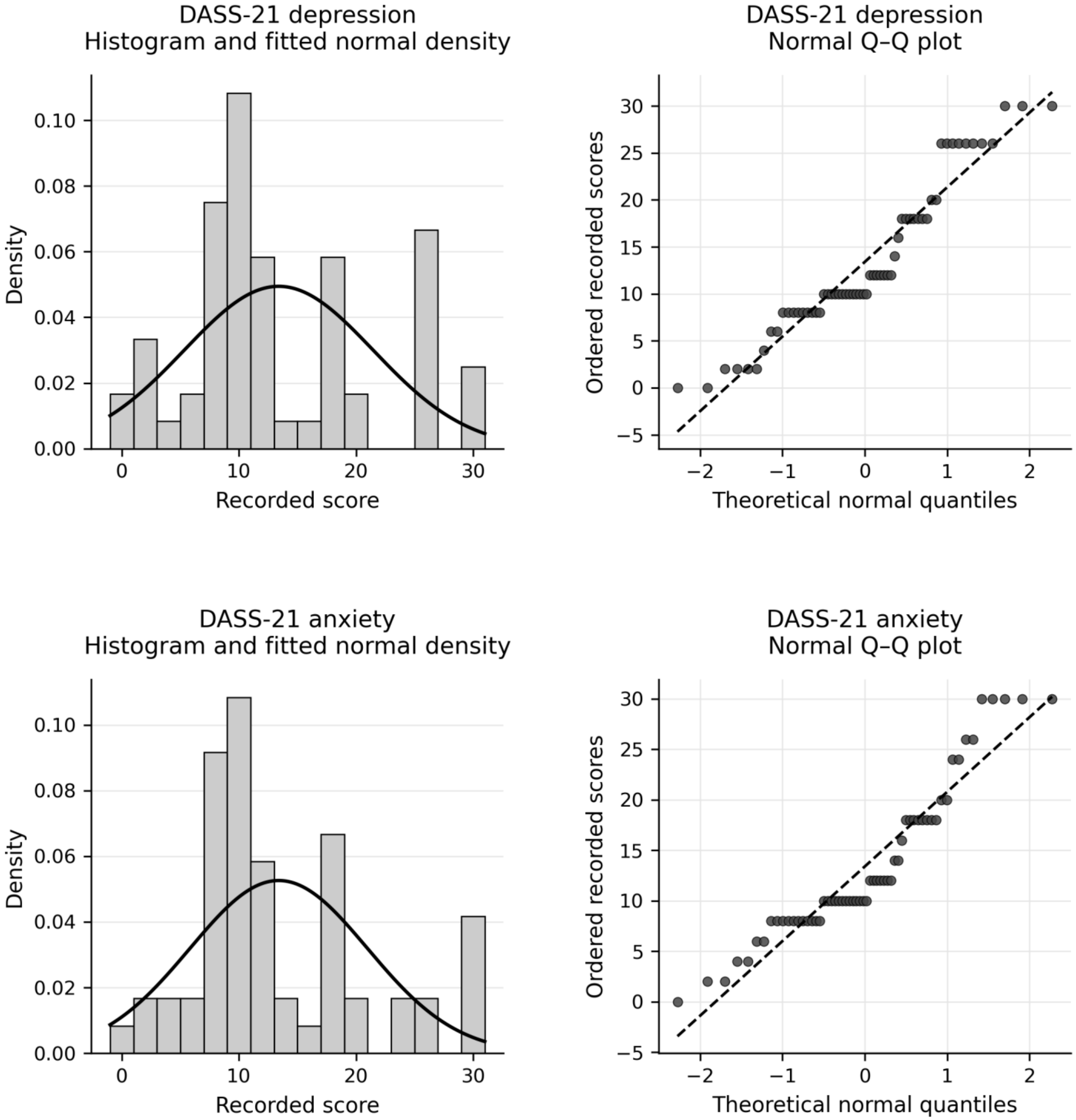

Figure S1. Baseline DASS-21 depression (top) and anxiety (bottom), n = 60. Histograms with fitted normal densities are on the left; normal Q–Q plots are on the right.

DASS 21 stress and kinesiophobia

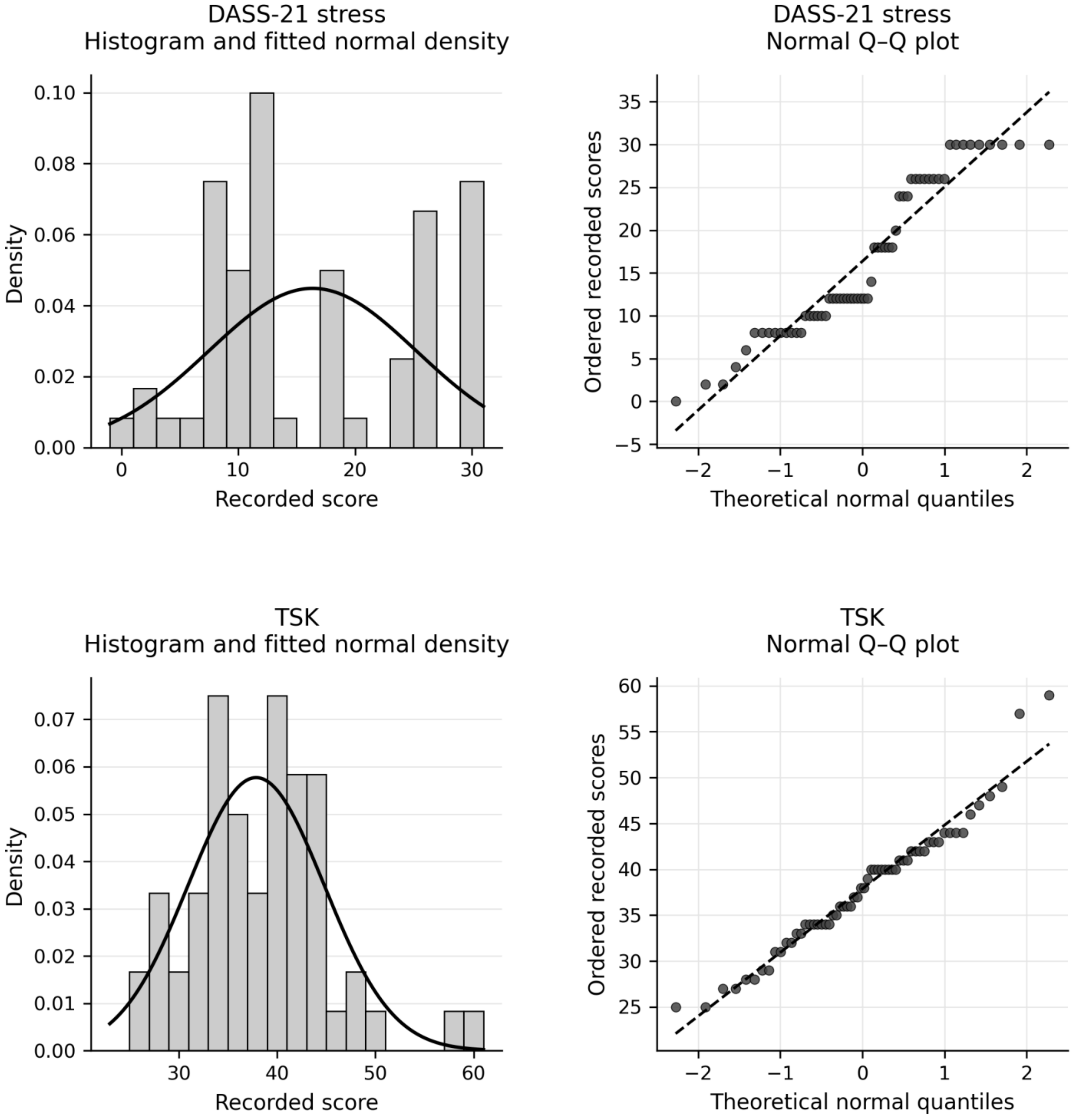

Figure S2. Baseline DASS-21 stress (top) and TSK (bottom), n = 60. Histograms with fitted normal densities are on the left; normal Q–Q plots are on the right.

Catastrophizing and Week 8 adherence

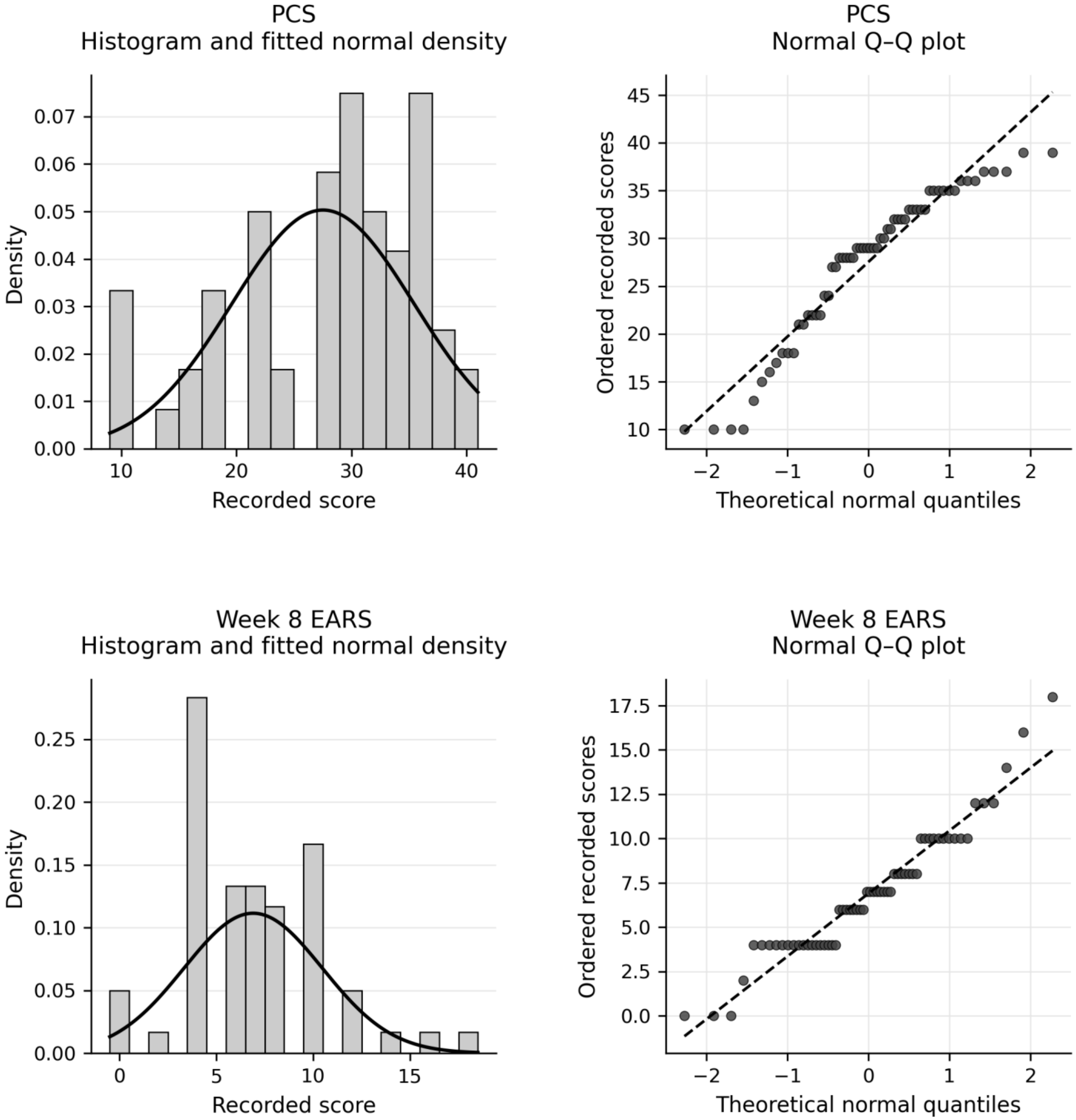

Figure S3. Baseline PCS (top) and Week 8 EARS (bottom), n = 60. Histograms with fitted normal densities are on the left; normal Q–Q plots are on the right.
